# Potentials and hurdles for human MRI and MRS experiments at 20 T

**DOI:** 10.1186/1532-429X-15-S1-W23

**Published:** 2013-01-30

**Authors:** T Budinger

**Affiliations:** 1 Bioengineering, Univ. of Calif. Berkeley, Berkeley, CA, USA

## Background

The successes in achieving nearly artifact-free images at proton frequencies of 300 - 500 MHz (7 to 11.7 T) in animals and the human brain underpin a quest for even higher fields for experimental medical science of relatively low gyromagnetic ratio nuclei in the range of 300 MHz at a field of 20 tesla.

The advantages for imaging intracellular and extracellular concentrations of quadraolar nuclei (e.g. Na, K, Cl) as well as the sensitivity increases for carbon-13, oxygen-17, and the expected spectral dispersion increases for carbon-13 and phosphorous-31 prompt us to pursue animal and human studies at 20 T.

## Methods

Methods for establishment of a National 20 T project include:

1. Statement of the unique medical science goals that can be achieved by a 65 cm diameter clear bore magnet with a homogeneous field of at least 1 ppm in a 16 cm diameter sphere.

2. Magnet technology capabilities needed to achieve a large bore homogeneous field at 20 T.

3. Physiological limitations and potential health hazards at

20 T and dB/dz associated with entrance and exit from this field.

## Results

Feasibility of measuring intra and extracellular sodium has been established by multiple quantum spectroscopy (Schepkin et al. 1996) and by relaxographic imaging (Labadie et al.1994); and for intra and extracellular potassium by Kuki et al (1990) in the isolated perfused heart. These studies were at fields much lower than 20 T. The compelling rationale for sodium, potassium and chloride ion measurements is the role they play in resting membrane potentials particularly of the brain and possibly in the etiology of in mental behavior. One can show that imaging resting membrane potential is feasible at 20 T with 5 mm resolution. Feasibility of imaging oxygen-17 has been established at 7 T by the Minnesota group (Zhu et al. 2005) Major advances can be predicted for in vivo measurements using chemical exchange saturation transfer.Major physiological effects are expected for perturbations of the vestibular apparatus from a combination of Lorentz and Faraday effects, but probably mostly from torques on assemblages with significant susceptibility anisotropy.Major magnet construction barriers are the volume forces that must be anticipated in structural materials as well as the coil materials. . Selection of high temperature superconductor material and possible hybrid design exercises are the next important step

## Conclusions

The medical science potentials for MRI and MRS at 20 T in human biochemistry, nutrition, neuroscience, cardiovascular science and oncology are beyond the capabilities of any other measurement systems because of the capabilities to measure the amount and identity of a broad spectrum of substances including identification of ions in the different environments in and outside the cell. The engineering challenges have no known impossible barriers, the health hazards can be evaluated cautiously, but the cost for a functional facility will approach $100 million.

